# Elucidating the Synergic Effect in Nanoscale MoS_2_/TiO_2_ Heterointerface for Na‐Ion Storage

**DOI:** 10.1002/advs.202204837

**Published:** 2022-10-30

**Authors:** Chunrong Ma, Dewen Hou, Jiali Jiang, Yanchen Fan, Xiang Li, Tianyi Li, Zifeng Ma, Haoxi Ben, Hui Xiong

**Affiliations:** ^1^ Key Laboratory of Bio‐Fibers and Eco‐Textiles Qingdao University Qingdao Shandong 266071 China; ^2^ Micron School of Materials Science and Engineering Boise State University Boise ID 83725 USA; ^3^ Center for Nanoscale Materials Argonne National Laboratory Lemont IL 60439 USA; ^4^ Shandong Key Laboratory of Water Pollution Control and Resource Reuse School of Environmental Science and Engineering Shandong University Qingdao Shandong 266237 China; ^5^ SUSTech Academy for Advanced Interdisciplinary Studies and Department of Materials Science & Engineering Southern University of Science and Technology Shenzhen Guangdong Province 518055 China; ^6^ Chemical Sciences and Engineering Division Argonne National Laboratory Lemont Illinois 60439 USA; ^7^ X‐Ray Science Division Argonne National Laboratory Lemont IL 60439 USA; ^8^ Shanghai Electrochemical Energy Devices Research Centre School of Chemistry and Chemical Engineering Shanghai Jiao Tong University Shanghai 200240 China; ^9^ Center for Advanced Energy Studies Idaho Falls 83401 USA

**Keywords:** fast charging, heterointerfaces, interfacial charge storage, intrinsic interfacial electric field effect, sodium ion batteries

## Abstract

Interface engineering in electrode materials is an attractive strategy for enhancing charge storage, enabling fast kinetics, and improving cycling stability for energy storage systems. Nevertheless, the performance improvement is usually ambiguously ascribed to the “synergetic effect”, the fundamental understanding toward the effect of the interface at molecular level in composite materials remains elusive. In this work, a well‐defined nanoscale MoS_2_/TiO_2_ interface is rationally designed by immobilizing TiO_2_ nanocrystals on MoS_2_ nanosheets. The role of heterostructure interface between TiO_2_ and MoS_2_ by operando synchrotron X‐ray diffraction (sXRD), solid‐state nuclear magnetic resonance, and density functional theory calculations is investigated. It is found that the existence of a hetero‐interfacial electric field can promote charge transfer kinetics. Based on operando sXRD, it is revealed that the heterostructure follows a solid‐solution reaction mechanism with small volume changes during cycling. As such, the electrode demonstrates ultrafast Na^+^ ions storage of 300 mAh g^−1^ at 10 A g^−1^ and excellent reversible capacity of 540 mAh g^−1^ at 0.2 A g^−1^. This work provides significant insights into understanding of heterostructure interface at molecular level, which suggests new strategies for creating unconventional nanocomposite electrode materials for energy storage systems.

## Graphical abstract

The heterointerfaces play an important role in providing additional active sites for enhanced charge storage as well as give rise to pseudocapacitive behavior with suppressed phase transitions for fast kinetics. The built‐in electrical field could reduce the diffusion barrier to strengthen the ion diffusion and charge transfer.

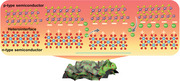

## 1 Introduction

Lithium‐ion batteries (LIBs) have garnered significant attention in the past decades and have been successfully implemented in portable electronics and electric vehicles because of their high energy and power densities. With the booming electric vehicle market, the limited lithium resources and the rising raw materials cost are the bottlenecks for the sustainable growth of large‐scale energy‐storage systems.^[^ ^1^, ^2^ ^]^ Sodium‐ion batteries (SIBs) are an attractive alternative for large‐scale energy storage systems in terms of the abundance of sodium resources and the low cost. Nevertheless, the sluggish reaction kinetics and severe volume change resulting from the larger ionic radius of Na^+^ currently hinder the realization of the high power density and the long‐term cycle life of SIBs.^[^ ^3^, ^4^, ^5^, ^6^ ^]^ Therefore, discovering and developing advanced electrode materials with fast ion diffusion and long lifespan is in urgent need to promote the practical application of SIBs. Among various anode materials explored in SIBs, two‐dimensional molybdenum disulfide (MoS_2_) has been extensively studied due to its high specific capacity (670 mAh g^−1^) and intrinsically enhanced safety.^[^ ^7^, ^8^, ^9^ ^]^ In particular, the large interlayer space of 0.62 nm and weak van der Waals interaction between layers are conductive to facilitate the reversible Na^+^ ions insertion/extraction. Nevertheless, the restacking agglomeration of 2D MoS_2_ layer during charge/discharge processes leads to continuous capacity fading.^[^ ^10^ ^]^ Additionally, the reaction intermediates (e.g., Na_2_S) generated from the conversion reaction could cause severe redox shuttle issues, which further exacerbates the capacity fading. To address these issues, tremendous efforts have been devoted. The major effort is related to engineering a variety of MoS_2_/carbon nanocomposites with tailored structures to minimize the mechanical stress/strain caused by volume change and to simultaneously enhance electronic conductivity.^[^ ^11^, ^12^, ^13^, ^14^, ^15^ ^]^ Despite many progresses made in the past, the partial agglomeration and sluggish kinetics resulting from the intrinsic poor electronic conductivity of MoS_2_ are still the paramount challenge for high‐performance SIBs.

Constructing heterostructure by interfacial design has been developed in many research fields, which could endow composites with unprecedented properties.^[^ ^16^, ^17^ ^]^ By reducing the length scale of the heterostructure interface down to the nanoscale regime, strong internal electrical field and increased grain boundaries may enhance the reaction kinetics and charge storage. Recent study confirms that polysulfides produced during a deep sodiated state can be tightly bonded to some oxides such as MgO, MnO, SnO_2_, and TiO_2_.^[^ ^18^, ^19^, ^20^, ^21^ ^]^ Among the various secondary phase materials, TiO_2_ is an attractive anode for SIBs due to its reactivity with Na^+^.^[^ ^22^, ^23^, ^24^ ^]^ Additionally, first‐principles calculations suggested that TiO_2_ shows a larger bonding energy (≈2.30 eV) with polysulfides than that of polysulfides on carbon (<0.1 eV),^[^ ^25^ ^]^ which could restrict the reaction with electrolyte and enhance the reversibility of the conversion reaction of MoS_2_. Therefore, it is reasonable that rationally integrate MoS_2_ with TiO_2_ may offer new prospects of the electrode for improving its electrochemical properties. Nevertheless, most previously reported heterostructures with MoS_2_ show dendritic/dumbbell‐like morphology,^[^ ^26^, ^27^, ^28^ ^]^ and the inferior chemical bonding between MoS_2_ and other metal oxides leads to undesirable structural instability and limits both electron and ion transport. It is known that, besides the enhanced reaction kinetics, interface engineering in electrode materials may increase electrode capacities that often exceed theoretical values.^[^ ^29^, ^30^, ^31^, ^32^ ^]^ Nevertheless, little is known regarding what contribute to the enhanced capacity and most work attribute the enhancement to the vaguely defined “synergistic effect”. The synergetic effect on the Na^+^ ions storage mechanism of the composite electrode remains unclear and the effect of heterointerface in the enhanced sodium ion storage has been often overlooked. More efforts are needed to be devoted to investigate the mechanisms of heterointerface toward enabling performance enhancement. Therefore, engineering stable heterostructure by rational design of MoS_2_/TiO_2_ hybrids can serve as a good model system to unveil the synergetic effect in composite electrode materials for SIBs.

Here, we rationally designed and synthesized a hierarchical MoS_2_/TiO_2_ heterostructure to study the synergetic effect of heterostructure interface, in which carbon‐coated 2D MoS_2_ nanosheets were decorated with ultrafine TiO_2_ nanoparticles on flexible carbon nanotubes (CNT‐MoS_2_/TiO_2_‐C). The unique structure provides a solution to mitigate the degradation issue of MoS_2_ anode for SIBs. The ultrafine TiO_2_ nanoparticles are tightly anchored on MoS_2_ nanosheets, which is assisted by the carbon shell coating through strong interfacial contact. The heterointerface is formed between MoS_2_ and TiO_2_, which results in a solid‐solution mechanism with lack of first‐order phase transitions often seen in bulk materials for charge storage to enhance the structural stability during cycling. Moreover, the well‐dispersed TiO_2_ nanoparticles can adsorb polysulfides and avoid the aggregation of MoS_2_ during the repeated charge/discharge processes, which effectively enhances the reaction reversibility of MoS_2_ at the interface. Furthermore, the strong coupling of MoS_2_ nanosheets and ultrafine TiO_2_ can provide additional sites for Na^+^ ions storage and promote the pseudocapacitive behavior, ensuring the ultrafast reaction kinetics. Importantly, solid‐state nuclear magnetic resonance (NMR) and first‐principles calculations provide an in‐depth understanding toward the Na^+^ ions transport behavior and charge storage mechanisms of the CNT‐MoS_2_/TiO_2_‐C electrode. The as‐prepared CNT‐MoS_2_/TiO_2_‐C demonstrates a high reversible capacity of 540 mAh g^−1^ at a current density of 0.2 A g^−1^, excellent rate capability, and long cycling stability for 5000 cycles at 5 A g^−1^.

## 2 Results and Discussion

**Figure 1:**
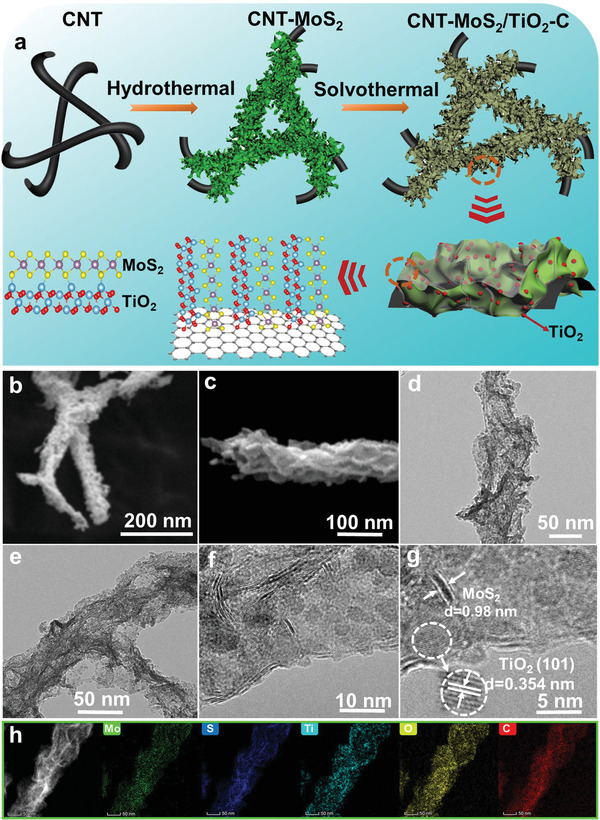
a) Illustration of the synthesis of CNT‐MoS_2_/TiO_2_‐C hybrids, b‐c) SEM image, d‐g) TEM image and h) EDX mapping with the corresponding area of the as‐prepared CNT‐MoS_2_/TiO_2_‐C.

The typical synthesis process of CNT‐MoS_2_/TiO_2_‐C is illustrated in **Figure** **1**a. Briefly, MoS_2_ nanosheets anchored on the CNT surface were synthesized by a hydrothermal process with Na_2_MoO_4_, thiourea, and CNT as the precursors. Subsequently, the ultrafine TiO_2_ nanoparticles were grown on MoS_2_ nanosheets by a solvothermal process. The morphology and structure of the CNT‐MoS_2_/TiO_2_‐C sample were characterized by scanning electron microscopy (SEM) and transmission electron microscopy (TEM). As shown in Figure 1b, the CNT‐MoS_2_/TiO_2_‐C sample demonstrates a typical 1D structure of CNT with an average diameter of ≈80 nm. The surface of CNT‐MoS_2_/TiO_2_‐C is coarser than that of pure CNT (Supporting Information, Figure S1) due to the existence of densely packed and wrinkled MoS_2_ nanosheets. The abundant voids enclosed by plate‐like structure with a thickness of ≈15 nm can be seen from SEM at high magnification (Figure 1c). This porous structure can effectively accommodate the volume expansion during cycling. TEM results (Figure 1d‐e) indicate the typical coaxial morphology of the sample, which suggest that the 2D MoS_2_ nanosheets were successfully grown on the CNT surface. The CNT served the role of a cable core to facilitate ion/electron transport. The high‐resolution TEM image (Figure 1f) illustrates the uniform decoration of TiO_2_ nanoparticles on MoS_2_ nanosheets, indicating the successful formation of MoS_2_/TiO_2_ nanocomposite structure. The ultrafine TiO_2_ nanoparticles were encapsulated within the carbon layer and this robust carbon shell can effectively inhibit the aggregation of MoS_2_ during repeated sodiation/desodiation processes. Meanwhile, the carbon coating can enhance the interface bonding strength between MoS_2_ and TiO_2_, ensuring structural stability. To further investigate the feature of CNT‐MoS_2_/TiO_2_‐C, the enlarged TEM image of a selected area in the same sample were shown in Figure 1g. The ultrafine TiO_2_ nanoparticles with a particle size of ≈4 nm are clearly visible and the lattice spacing of 0.354 nm can be ascribed to the (101) plane of anatase TiO_2_. Such small size can facilitate the pseudocapacitive storage for high rate capability. TEM results demonstrate the few‐layered MoS_2_ nanosheets with an expanded interlayer spacing of 0.98 nm. Energy dispersive X‐ray spectroscopy (EDX) mapping was performed to investigate the elemental distribution of CNT‐MoS_2_/TiO_2_‐C (Figure 1h). It shows that the elements of Mo, S, Ti, O, and C are uniformly distributed within the nanocomposite.

**Figure 2:**
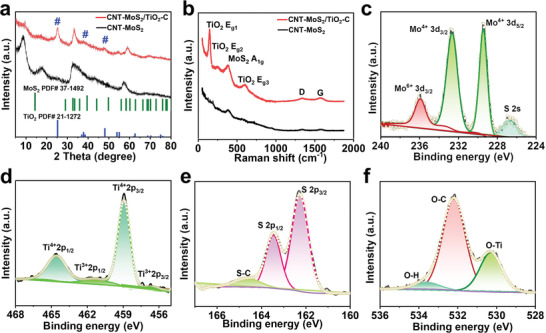
a) XRD patterns of CNT‐MoS_2_/TiO_2_‐C and CNT‐MoS_2_, b) Raman spectra of CNT‐MoS_2_/TiO_2_‐C and CNT‐MoS_2_ and c–f) High‐resolution XPS spectra of the Mo 3d, Ti 2p, S 2p, and O 1s in the CNT‐MoS_2_/TiO_2_‐C.

The structure and crystallinity of the as‐prepared CNT‐MoS_2_/TiO_2_‐C are characterized by X‐ray powder diffraction (XRD) and Raman spectroscopy. The XRD pattern of CNT‐MoS_2_/TiO_2_‐C is shown in **Figure** **2**a. The (002) characteristic peak of MoS_2_ at 13.9^o^ is absent and two new peaks at 8.7^o^ and 17.5^o^ are observed, which indicates the existence of few‐layers MoS_2_.^[^ ^33^, ^34^ ^]^ According to Bragg's Law (2*d*sin*θ* = *nλ*),^[^ ^35^ ^]^ the expanded interlayer distance of 0.98 nm (8.7^o^) is determined. Meanwhile, the characteristic peaks of TiO_2_ can be seen at 25.3^o^, 38.5^o^, and 48.2^o^, which are attributed to the (101), (112), and (200) planes of anatase TiO_2_ (JCPDS No. 21–1272). The Raman spectra of CNT‐MoS_2_/TiO_2_‐C are displayed in Figure 2b. The peaks located at 145, 209, and 639 cm^−1^ match well with the *E*_g(1),_ *E*_g(2)_, and *E*_g(3)_ modes of anatase TiO_2_, respectively. Moreover, the MoS_2_ characteristic peak at 465 cm^−1^ is present. Two peaks at 1354 and 1597 cm^−1^ are ascribed to the D‐ (induced by defects in carbon) and G‐band (representative of graphitic structure), typical of carbonaceous materials.^[^ ^26^ ^]^ The intensity ratio of D and G bands is 1.14, suggesting that the carbon is disordered in the CNT‐MoS_2_/TiO_2_‐C composite. The surface and chemical properties of the as‐prepared CNT‐MoS_2_/TiO_2_‐C sample were investigated by X‐ray photoelectron spectroscopy (XPS). XPS survey scan is shown in Figure S2a (Supporting Information). From the high‐resolution Mo 3d XPS spectrum (Figure 2c), the peaks centered at 229.2 and 232.8 eV can be attributed to the Mo^4+^ 3d_5/2_ and Mo^4+^ 3d_3/2_, respectively. Notably, an additional peak appears at 236 eV, which can be attributed to the Mo^6+^ 3d_3/2_ due to the formation of MoS_2_ in the air.^[^ ^15^ ^]^ The high‐resolution XPS spectrum of Ti 2p in Figure 2d shows two peaks at binding energy of 458.8 and 464.7 eV, which are assigned to the Ti^4+^ 2p_3/2_ and Ti^4+^ 2p_1/2_, respectively. In addition, the small peaks at binding energy of 457.3 eV and 461.8 eV are assigned to Ti^3+^ 2p_3/2_ and Ti^3+^ 2p_1/2_, respectively, indicating the existence of Ti^3+^ in CNT‐MoS_2_/TiO_2_‐C.^[^ ^36^ ^]^ For the high‐resolution spectrum of S 2p (Figure 2e), it can be deconvoluted into three peaks with binding energy of 162.1, 163.5, and 164.7 eV, which are attributed to the S 2p_3/2_, S 2p_1/2,_ and S–C bonds, respectively.^[^ ^37^ ^]^ The O 1s high‐resolution spectrum can be fitted into three component peaks as shown in Figure 2f. The three peaks at binding energy of 530.2, 532.3, and 533.3 eV correspond to the Ti–O, C–O, and O–H, respectively.^[^ ^38^ ^]^ The C 1s spectrum (Figure S2b, Supporting Information) is resolved into four peaks at 284.3, 285.1, 285.2, and 290.2 eV, respectively. The strong peak at 284.3 eV can be assigned to the C–C, the latter three peaks are corresponding to the C–O, C=O, and O–C=O, respectively.^[^ ^39^ ^]^ The thermodynamic stability of CNT‐MoS_2_/TiO_2_‐C when exposed in the atmosphere was studied by thermogravimetric analysis (TGA). As shown in Figure S3 (Supporting Information), the CNT‐MoS_2_/TiO_2_‐C possesses good thermal stability when the temperature is below 200 °C. And the calculated MoS_2_/TiO_2_ content in the CNT‐MoS_2_/TiO_2_‐C is 89.3%.

**Figure 3:**
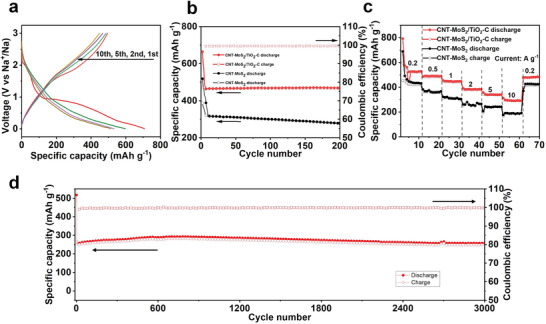
a) Charge‐discharge profiles of CNT‐MoS_2_/TiO_2_‐C electrode at a current density of 0.2 A g^−1^, b) Cycling performance at a current density of 1 A g^−1^, c) Rate capability at various current density from 0.2 to 10 A g^−1^, and d) Long‐term cyclic stability of CNT‐MoS_2_/TiO_2_‐C electrode at a current density of 5 A g^−1^.

The galvanostatic charge‐discharge profiles of the CNT‐MoS_2_/TiO_2_‐C electrode at a current density of 0.2 A g^−1^ are shown in **Figure** **3**a. During the first cycle, the CNT‐MoS_2_/TiO_2_‐C electrode delivers discharge and charge specific capacity of 708 mAh g^‐1^ and 543 mAh g^−1^, respectively, corresponding to a high initial Coulombic efficiency (ICE) of 77%. The initial capacity loss can be attributed to the irreversible reaction arising from the formation of solid electrolyte interphase (SEI) layer.^[^ ^10^, ^11^, ^12^, ^13^ ^]^ In contrast, the discharge and charge specific capacity of CNT‐MoS_2_ electrode is only 623 and 424 mAh g^−1^ with an ICE of 68.2% (Figure S4, Supporting Information). The enhanced ICE in CNT‐MoS_2_/TiO_2_‐C electrode may be attributed to the stabilization of the heterointerface during cycling. The introduced TiO_2_ nanocrystals on the MoS_2_ nanosheets could adsorb polysulfides to enhance the reversibility of reaction. In addition, the interfaces between CNT and amorphous carbon could provide more active sites for Na^+^ ions.^[^ ^40^ ^]^ From the second cycle, the CNT‐MoS_2_/TiO_2_‐C electrode displays a high reversible specific capacity of 523 mAh g^−1^ and the CE quickly reaches 90%. In the following cycles, the CE of CNT‐MoS_2_/TiO_2_‐C electrode keeps increasing gradually, and the discharge capacity maintains at ≈520 mAh g^−1^. The cycling performance of the samples is also evaluated at a current density of 1 A g^−1^ and the result is shown in Figure 3b. The CNT‐MoS_2_/TiO_2_‐C electrode retains its initial capacity even after 200 cycles with the Coulombic efficiency close to 100%. In contrast, CNT‐MoS_2_ electrode fades quickly — only 303 mAh g^−1^ is retained for after 200 cycles at current density of 0.2 A g^−1^. The poor cycling stability of CNT‐MoS_2_ electrode is ascribed to the structure collapse and the reduction of active sites due to the aggregation of MoS_2_ nanosheets during the charge/discharge processes (Figure S5, Supporting Information).

The rate capability of the CNT‐MoS_2_/TiO_2_‐C electrode as well as CNT‐MoS_2_ electrode are evaluated at various current density from 0.5 to 10 A g^−1^. As shown in Figure 3c, the CNT‐MoS_2_/TiO_2_‐C electrode exhibits capacities of 540, 500, 460, 410, and 360 mAh g^−1^ at the current densities of 0.2, 0.5, 1.0, 2.0, and 5.0 A g^−1^, respectively. In addition, it maintains a high discharge capacity up to 300 mA h g^−1^ even at a high current density of 10 A g^−1^. After deep charge/discharge at high current rates, the discharge capacity of CNT‐MoS_2_/TiO_2_‐C electrode returns to 500 mAh g^−1^ when the current is ramped back to 0.2 A g^−1^. The rate capability in CNT‐MoS_2_/TiO_2_‐C electrode especially at ultra‐fast charge/discharge demonstrates the stable structure of the electrode. Additionally, the rate performance of CNT‐MoS_2_ electrode is shown as a comparison. It exhibits low discharge capacity of 410, 350, 300, 250, and 200 mAh g^−1^ at the current density of 0.2, 0.5, 1.0, 2.0, and 5.0 A g^−1^, respectively. These values are lower than those of CNT‐MoS_2_/TiO_2_‐C electrode. The rate capability of bare TiO_2_ is shown in Figure S6 (Supporting Information), which only exhibits ≈70 mAh g^−1^ at the current rate of 0.5 A g^−1^, and the discharge capacity plunges sharply with the increase of the current rate. Apparently, the rate capability in CNT‐MoS_2_/TiO_2_‐C electrode is superior as compared to CNT‐MoS_2_ or CNT‐TiO_2_ electrode. The electrochemical performance of CNT‐MoS_2_/TiO_2_‐C electrode is compared with reported works, the results is shown in FigureS7 (Supporting Information).

The long‐term cycling stability is important for the SIBs. However, the structural collapse and slow reaction kinetics resulting from the large radius of Na^+^ ions significantly hinder the Na^+^ ions insertion/extraction in electrode materials. The cycling performance of CNT‐MoS_2_/TiO_2_‐C electrode is evaluated at a high current rate of 5 A g^−1^ (Figure 3d). A high capacity of 273 mAh g^−1^ still remains after 3000 cycles, indicating the superior cycling stability of the CNT‐MoS_2_/TiO_2_‐C electrode. Moreover, the Columbic efficiency reaches to 100% at the 8th cycle, which suggests the high reversibility of the electrode.

**Figure 4:**
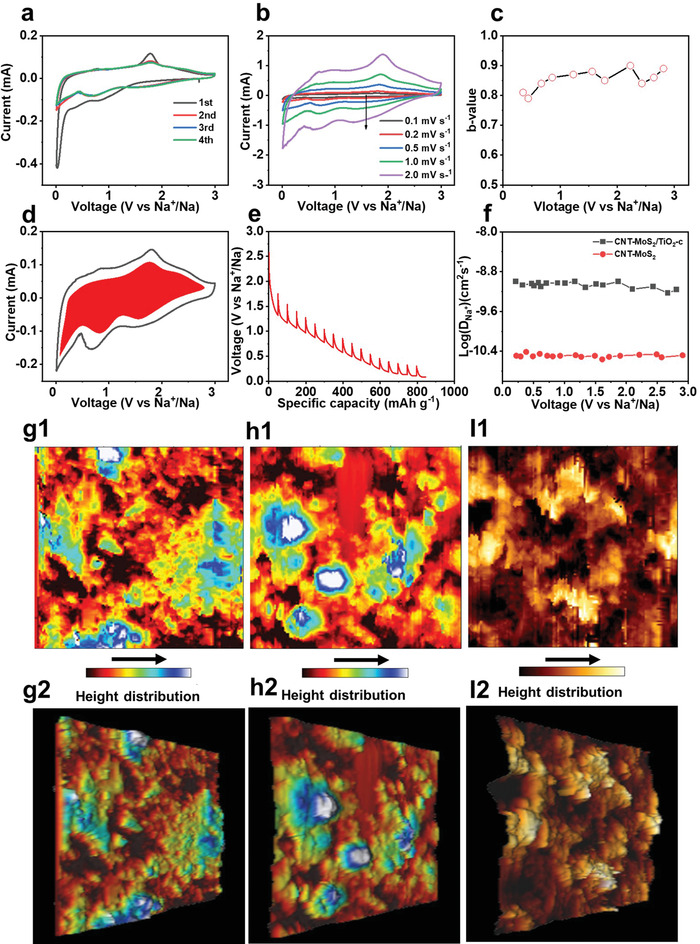
a) CV curves at scan rate of 0.1 mV s^−1^, b) CV curves at various scan rate from 0.1 to 2.0 mV s^−1^, c) the *b*‐value at different voltages, d) the capacitive contribution at scan rate of 0.2 mV s^−1^, e) GITT profile, f) Na^+^ ions diffusion coefficient at corresponding potentials, AFM images g1) 2D image, and g2) 3D image of fresh CNT‐MoS_2_/TiO_2_‐C, h1) 2D image, and h2) 3D image of CNT‐MoS_2_/TiO_2_‐C electrode discharged to 0.01 V, I1) 2D image, and I2) 3D image of CNT‐MoS_2_ electrode discharged to 0.01 V.

The Na^+^ ions storage mechanism of CNT‐MoS_2_/TiO_2_‐C is investigated via cyclic voltammetry (CV). **Figure** **4**a shows the CV curves of CNT‐MoS_2_/TiO_2_‐C electrode during the initial four cycles at a scan rate of 0.1 mV s^−1^. In the first cathodic scan, a peak at 0.8 V can be observed, which is attributed to the insertion of Na^+^ ions in MoS_2_: MoS_2_ + *x*Na^+^ + *x*e^−^ → Na_*x*_MoS_2_, as well as the formation of SEI layer.^[^ ^41^ ^]^ The reduction peak located at 0.5 V corresponds to the conversion reaction from Na*_x_* MoS_2_ to Mo: Na_*x*_MoS_2_ + (4 − *x*)Na^+^ + (4 − *x*)e^−^ → Mo + 2Na_2_S.^[^ ^42^ ^]^ For the initial anodic scan, the sharp peak at 1.75 V is ascribed to the Na^+^ ions extraction and Mo oxidation: Mo + 2Na_2_S → MoS_2_ + 4Na^+^ + 4e^−^.^[^ ^42^ ^]^ For the subsequent cycles, the shape of CVs is well maintained without obvious change and overlapped together, suggesting the high reversibility of CNT‐MoS_2_/TiO_2_‐C electrode. To better understand the kinetics of Na^+^ ions storage in CNT‐MoS_2_/TiO_2_‐C electrode, CV curves were measured at various scan rates from 0.1 to 2 mV s^−1^ (Figure 4b). The charge storage mechanism can be described by the power law relationship: *i = av^b^*,^[^ ^43^ ^]^ where *i* is the current density, *v* is the scan rate and *a* and *b* are adjustable constants. Particularly, the *b* value is in the range of 0.5 to 1, which can be determined from the slope of log *i* versus log *v* plots. The *b* value of 0.5 indicates that the charge storage process is controlled by diffusion, while the *b* value of 1.0 represents the capacitive behavior. Figure 4c shows *b* value at corresponding potentials during the cathodic process, in which all of the values are almost above 0.8. Such results indicate that the Na^+^ ions storage in CNT‐MoS_2_/TiO_2_‐C electrode has a high contribution from pseudocapacitance, resulting in fast kinetics.

To quantitatively determine the capacitive contribution in the whole charge process, the current can be expressed by the equation: *i =* k_1_ *v+*k_2_ *v^1/2^*,^[^ ^43^ ^]^ where the *k_1_v* is the capacitive contribution and the *k_2_v^1/2^* is the diffusion‐controlled process. The capacitive contribution in the entire CV profile at scan rate of 0.2 mV s^−1^ is shown in Figure 4d. About 75% charge storage is from the pseudocapacitive process (red area), and the diffusion behavior mainly occurs near the redox peaks. Based on the CV analysis, it is concluded that the fast reaction kinetics for the ultra‐fast rate capability of the CNT‐MoS_2_/TiO_2_‐C electrode are mainly originated from its pseudocapacitive characteristics. Galvanostatic intermittent titration technique (GITT, Figure S8, Supporting Information) was conducted to further investigate the Na^+^ ions diffusion coefficient (D_Na_^+^). Figure 4e illustrates the GITT profile of CNT‐MoS_2_/TiO_2_‐C electrode obtained by applying a series of current pulses at 100 mA g^−1^ for 0.5 h at 2 h rest intervals. The D_Na_^+^ can be calculated according to the equation^[^ ^44^ ^]^:

(1) $$D_{\mathrm{Na}+}=\frac{4}{\pi }{\left(\frac{m_{B}V_{M}}{M_{B}A}\right)}^{2}{\left(\frac{\Delta E_{s}}{\Delta E_{t}}\right)}^{2}\left(\tau \ll \frac{L^{2}}{D_{\mathrm{Na}+}}\right)$$

As shown in Figure 4f, the D_Na_^+^ of the CNT‐MoS_2_/TiO_2_‐C electrode is mostly constant at ≈10^−9^ cm^2^ s^−1^ across the voltage range in the discharge process. In contrast to the CNT‐MoS_2_ electrode, CNT‐MoS_2_/TiO_2_‐C electrode demonstrates improved diffusion kinetic.

Based on the aforementioned results, the excellent electrochemical performance of CNT‐MoS_2_/TiO_2_‐C electrode can be ascribed to the following factors: 1) The ultrafine TiO_2_ nanoparticles on MoS_2_ nanosheets can effectively suppress the aggregation of MoS_2_ restacking during Na^+^ ions insertion/extraction. Meanwhile, the MoS_2_‐based anodes for SIBs often suffer from the shuttle effect of intermediate polysulfides during the charge/discharge process. The TiO_2_ nanoparticles uniformly distributed on the surface of MoS_2_ nanosheets serve as adsorbent to immobilize polysulfides, which effectively suppresses the shuttle of polysulfides for improved stability. 2) The expanded layer spacing of MoS_2_ nanosheets from 0.64 nm to 0.98 nm could not only facilitate the insertion and diffusion of Na^+^ ions but also offer more active sites to adsorb Na^+^ ions. It results in the enhanced kinetics of Na^+^ ions transport. Moreover, the voids enclosed by nanosheet arrays at the substrate surface could accommodate volume expansion. In order to investigate the surface feature of CNT‐MoS_2_/TiO_2_‐C electrode, the 3D rough reconstruction is conducted by atomic force microscope (AFM). As shown in Figure 4g1–2, the surface of the CNT‐MoS_2_/TiO_2_‐C electrode is relatively flat before discharging. After discharge to 0.01 V (Figure 4h1‐h2), the insertion of Na^+^ ions leads to a slightly coarse surface and expanded volume of the surface of CNT‐MoS_2_/TiO_2_‐C electrode. In contrast, the surface roughness of CNT‐MoS_2_ electrode (Figure 4I1‐2) significantly increases after being discharged to 0.01 V. 3) The glucose can not only serve as carbon source to improve the conductivity of the composites, but also its derived carbon layer acts as a protective layer to strengthen the heterointerface between MoS_2_ and TiO_2_ to avoid the structural collapse, both of which enhance the structural integrity and cycling stability. 4) The enhanced pseudocapacitance and hierarchical architectures can facilitate the kinetics in charge storage and transport and accommodate the volume expansion, which contributes to high rate capability.

**Figure 5:**
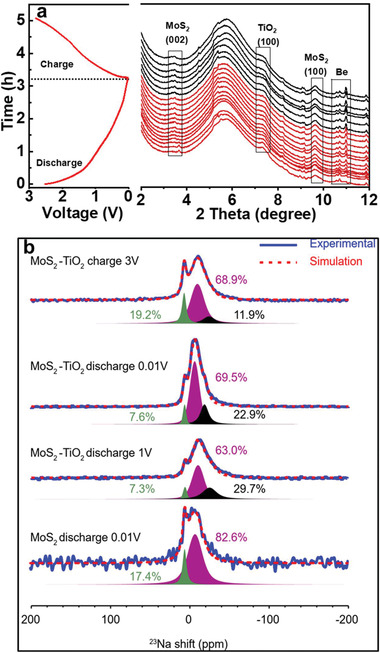
a) Operando sXRD patterns of as‐prepared CNT‐MoS_2_/TiO_2_‐C electrode and corresponding charge‐discharge profile of the first cycle, and b) Experimental and simulated ^23^Na NMR spectra of MoS_2_‐TiO_2_ and MoS_2_ electrodes at different states of charge.

In addition to features discussed above for enhancing SIB storage properties, the most crucial features of CNT‐MoS_2_/TiO_2_‐C electrode are that it offers abundant heterointerfaces to maximize the Na^+^ ions storage as well as provide stability during extended cycling. It is crucial to elucidate the effect of the heterointerface in the CNT‐MoS_2_/TiO_2_‐C electrode for its enhanced electrochemical performance. Operando sXRD was performed to monitor structural evolution of the CNT‐MoS_2_/TiO_2_‐C electrode during the charge/discharge process. The XRD patterns at different charge/discharge states during the first cycle were displayed in **Figure** **5**a with the corresponding charge/discharge profile of the first cycle between 0.01‐3 V shown in Figure 5b. The peaks located at 3.7^o^, 7.3^o^, and 9.6^o^ in the operando XRD are associated with the (002) plane of MoS_2_, (100) plane of anatase TiO_2_, and (100) plane of MoS_2_. During the Na^+^ ions intercalation process, the (002) peak of MoS_2_ progressively shifts to lower angles until 1.5 V, which is associated with the expansion of interlayer spacing. No new peaks appear in the following discharge process, indicating that the crystal structure is preserved and Na^+^ insertion into MoS_2_ occurs via intercalation into the layers following a solid‐solution mechanism. This result suggests that the Na^+^ storage mechanism in CNT‐MoS_2_/TiO_2_‐C electrode is lack of first‐order phase transition and follows a solid‐solution mechanism rather than the commonly‐discussed “decomposition” reaction.^[^ ^45^, ^46^ ^]^ The result is consistent with the pseudocapacitive behavior of the CNT‐MoS_2_/TiO_2_‐C electrode discussed earlier. It is postulated that such pseudocapacitive behavior occurs upon a solid‐solution phase transition process facilitated by the heterointerface between MoS_2_ and TiO_2_, which promote excellent structural stability during Na^+^ ion intercalation. Upon deintercalation, the crystal structure of MoS_2_ maintains well, suggesting that there is no significant volume change in the CNT‐MoS_2_/TiO_2_‐C electrode and it can reversibly accept/release Na^+^. The solid‐state nuclear magnetic resonance (NMR) was performed to evaluate Na^+^ storage in MoS_2_ and TiO_2_ of electrodes at different states of charge. Figure 5c shows the experimental and corresponding simulated ^23^Na‐NMR spectra of the CNT‐MoS_2_ electrode discharged to 0.01 V. From the simulated results, it can be concluded that a large amount of Na^+^ is inserted into MoS_2_ nanosheets when discharged to 0.01 V. The peak located at ‐6 ppm is ascribed to the Na^+^ inserted into MoS_2_, while the other resonance ≈6.8 ppm corresponds to SEI arising from the electrolyte decomposition at the first discharge process.^[^ ^47^ ^]^ In addition to the SEI peak at 6 ppm and Na in MoS_2_ nanosheets at ‐10 ppm, a new resonance at ‐25 ppm is detected in the spectra of the CNT‐MoS_2_/TiO_2_‐C electrode discharged to 1 V, which can be attributed to the Na^+^ stored at the interface between MoS_2_ and TiO_2_. According to the quantitative analysis, the content of Na in MoS_2_ and SEI is 63% and 7.3%, respectively. The rest of Na (29.7%) distributes at the interface between MoS_2_ and TiO_2_. For the sample discharged to 0.01 V, the ^23^Na resonance of Na in MoS_2_ demonstrates a shift from ‐10 ppm to ‐6.1 ppm due to the phase transformation of MoS_2_ during the discharge process. Quantitative analysis shows that 22.9% discharge capacity is from the interface when discharged to 0.01 V. It is worth noting that Na^+^ in TiO_2_ is not determined in the analysis. This is because the ^23^Na resonance of TiO_2_ is located at ‐7 ppm which overlaps with Na in MoS_2_.^[^ ^47^ ^]^ Therefore, it is difficult to distinguish it from the ^23^Na resonance of Na in MoS_2_ due to the weak intensity of Na in TiO_2_. When the electrode is charged (desodiated) to 3 V, the peak of Na in MoS_2_ can return to ‐10 ppm, indicating the reversibility of the Na insertion/extraction from MoS_2_ sheets. Such results reveal that the higher specific capacity of the CNT‐MoS_2_/TiO_2_‐C electrode is associated with not only the MoS_2_ and TiO_2_, but also the heterointerface that offers active sites for additional charge storage.

**Figure 6:**
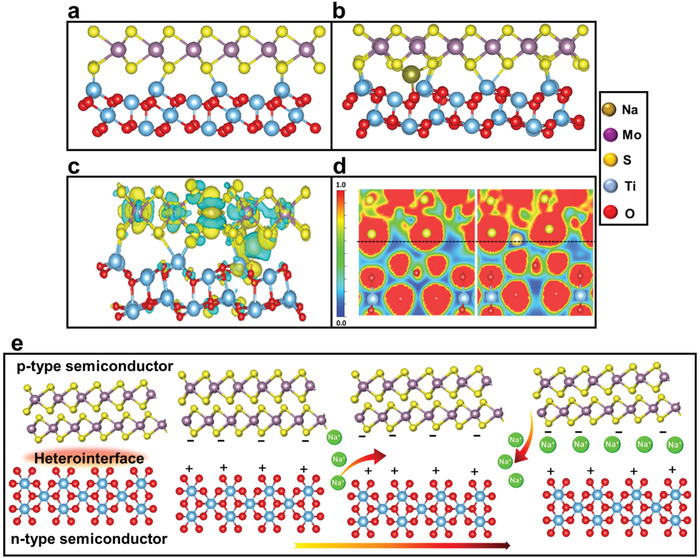
a) Relaxed MoS_2_/TiO_2_ interface structure, b) Na atom adsorption at the interface, c) Charge density difference plot, d) ELF plot after Na doping, and e) scheme illustration of Na^+^ ions storage mechanism for CNT‐MoS_2_/TiO_2_‐C electrode.

DFT calculations were performed to understand the interfacial behavior in the discharge process. The theoretical study offers an atomic level understanding of the improved electrochemical performance of CNT‐MoS_2_/TiO_2_‐C electrode. The models of MoS_2_ (001)‐TiO_2_ (110) heterojunction were conducted due to their similar orientation (**Figure** **6**a), which has been fully optimized. Before contact, MoS_2_ as a p‐type semiconductor exhibits the band gap of 1.89 eV,^[^ ^48^ ^]^ which is higher than that of TiO_2_ (n‐type semiconductor, 3.2 eV).^[^ ^49^ ^]^ To illustrate the activity of MoS_2_/TiO_2_ interface, the adsorption of Na^+^ on the MoS_2_, TiO_2_, and heterointerface were examined (Figure 6b and Figure S9, Supporting Information), respectively. Based on the optimizing model, the Na ions tend to coordinate with four nearest neighbor S atoms from MoS_2_ and also coordinate with one O atom at TiO_2_ surface with the Na–S and Na–O bonds, respectively. The results demonstrate that the adsorption energy at the heterointerface is larger than that at the separated MoS_2_ or TiO_2_ surface. The enhanced adsorption strength in MoS_2_/TiO_2_ interface suggests smaller energy barriers, which can be attributed to the synergetic effect. The heterointerface could offer additional affinity toward Na, which does not affect the original Na–S sites. The electronic structure analysis was performed to understand the synergetic behavior. The charge density difference at the interface with inserted Na is shown in Figure 6c, the charge redistribution mainly occurs at the interface where the charges accumulate around S and O atoms. It illustrates that the Na atom tend to donate charge to the surrounding S and O atoms during the sodiation process, which enhances the binding ability. This improvement can be qualitatively calculated by Bader charge analysis, which shows that doped Na atom contribute 1 eV to the S and O atoms. Moreover, the electron localization function (ELF) was conducted to explore the essence of interaction (Figure 6d). The charge density changes during Na insertion and the blue area around Na atom becomes dark while the red color increase around O and S atoms. ELF value distribution results illustrate that Na atoms interacts electrostatically with O and S atoms on the interface by yielding charges. It provides convincing evidence to support the charge separation at the heterointerface. Based on the above calculations, a possible Na^+^ ions storage mechanism is proposed in Figure 6e. The built‐in electric field at the heterointerface will form once the MoS_2_ and TiO_2_ are in contact, which will facilitate the accumulation of negative charges on the MoS_2_ side and positive charges on the TiO_2_ side. The negative charges accumulating at the MoS_2_ surface can be neutralized by attracting Na^+^ ions under the electric field, which greatly promotes the Na^+^ ions diffusion and enables the formation of a “sodium reservoir” in the heterostructure of the sample.

## 3 Conclusion

In summary, we designed and synthesized a novel heterointerface which is composed of amorphous carbon‐coated MoS_2_/TiO_2_ heterostructure anchored directly on CNT. The ultrathin MoS_2_ nanosheets are decorated with ultrafine TiO_2_ nanoparticles encapsulated in amorphous carbon and preferentially assemble into the sandwich‐like structure between CNT and the carbon outer layer. Such coaxial‐shaped structure constructed from CNT could offer facile paths for Na^+^ ions diffusion and improve structural stability. Particularly, the ultrafine particles can create abundant interfacial contacts between MoS_2_ nanosheets and TiO_2_ nanoparticles, which enhances the reaction kinetics, the structural stability, and modulates the electronic properties of MoS_2_. The experimental and theoretical results reveal that the abundant molecular scale interfacial sites play an important role in providing additional active sites for enhanced capacity. Moreover, the built‐in electrical field formed at the interface could reduce the diffusion barrier to strengthen the ion diffusion and charge transfer. Further, ultrafine TiO_2_ nanoparticles serve as an anchor to prevent MoS_2_ from restacking and aggregation as well as absorbents to bind polysulfide intermediates from redox shuttle for improved cycling stability. More importantly, the heterointerface between MoS_2_ and TiO_2_ gives rise to the pseudocapacitive behavior with suppressed first‐order phase transition during cycling for fast kinetics. Based on the strong interface synergistic effect, the CNT‐MoS_2_/TiO_2_‐C electrode demonstrates excellent rate capability (300 mAh g^−1^ at 10 A g^−1^) and superior cycling stability (over 3000 cycles was achieved at 5 A g^−1^). Given the insights obtained through the systematic study of the heterointerface in this work, it provides a new strategy to develop electrode materials with tailored interface with both high energy and power density for sodium ion storage.

## Conflict of Interest

The authors declare no conflict of interest.

## Supporting information

Supporting Information

## Data Availability

The data that support the findings of this study are available from the corresponding author upon reasonable request.
