# Supplementary material for: Elucidating the Synergic Effect in Nanoscale MoS2/TiO2 Heterointerface for Na‐Ion Storage
Source: Adv Sci (Weinh). 2022 Oct 30;9(35):2204837. doi: 10.1002/advs.202204837 (PMC9762294; doi:10.1002/advs.202204837)
Supplement: Supplementary file 1 — Supporting Information [file ADVS-9-2204837-s001.pdf]

ADVANCED SCIENCE
Open Access

Supporting Information

for *Adv. Sci.*, DOI 10.1002/advs.202204837

Elucidating the Synergic Effect in Nanoscale MoS_2/TiO_2 Heterointerface for Na-Ion Storage

Chunrong Ma, Dewen Hou, Jiali Jiang, Yanchen Fan, Xiang Li, Tianyi Li, Zifeng Ma, Haoxi Ben and Hui Xiong**

Elucidating the Synergic Effect in Nanoscale MoS_2/TiO_2 Heterointerface for Na-ion Storage

Chunrong Ma[1*], Dewen Hou[2,3], Jiali Jiang[4], Yanchen Fan[5], Xiang Li[6], Tianyi Li[7], Zifeng Ma[8], Haoxi Ben[1*], and Hui Xiong[2,9*]

[1]Key Laboratory of Bio-Fibers and Eco-Textiles, Qingdao University, Qingdao Shandong 266071, China

[2]Micron School of Materials Science and Engineering, Boise State University, Boise, ID 83725, United States

[3] Center for Nanoscale Materials, Argonne National Laboratory, Lemont, IL, 60439, United States

[4]Shandong Key Laboratory of Water Pollution Control and Resource Reuse, School of Environmental Science and Engineering, Shandong University, Qingdao, Shandong 266237, China

[5]SUSTech Academy for Advanced Interdisciplinary Studies and Department of Materials Science & Engineering, Southern University of Science and Technology, Shenzhen, Guangdong Province 518055, China

[6]Chemical Sciences and Engineering Division, Argonne National Laboratory, Lemont, Illinois 60439, United States

[7]X-Ray Science Division, Argonne National Laboratory, Lemont, IL, 60439, United States

[8]Shanghai Electrochemical Energy Devices Research Centre, School of Chemistry and Chemical Engineering, Shanghai Jiao Tong University, Shanghai, 200240, China.

[9]Center for Advanced Energy Studies, Idaho Falls, USA, 83401

*E-mail: clairexiong@boisestate.edu; mcrqust@126.com (corresponding authors)

Methods

Materials Synthesis: Firstly, the CNT-MoS_2 precursor was synthesized using a hydrothermal method. The mixture of 0.2 g Sodium Molybdenum Oxide Dihydrate ($Na_2MoO_4 \cdot 2H_2O$), 0.4g Thiourea (CH_4N_2S), 0.05 g Hexadecyl trimethyl ammonium Bromide (CTAB) and 30 mg acid-multiwalled carbon nanotubes (CNT) were mixed under ultrasonication for 30 min. The resulting solution was transferred to a Teflon-lined stainless steel autoclave and heated to 200 ^{o}C for 24 h. After cooling, the products were washed with ethanol and water several times. Secondly, the as-prepared CNT-MoS_2 (0.4 g) was added to 80 ml ethanol, then 0.3 ml tetrabutyl titanate and 0.2 g glucose were poured into the above solution under stir for 20 min. Subsequently, the mixed solution was transferred into a Teflon-lined stainless steel autoclave and heated to 170 ^{o}C for 12 h. The as-obtained products were centrifuged and pyrolyzed at 700 ^{o}C for 2h under Ar conditions. To compare the performance, control CNT-MoS_2 and TiO_2-C samples were synthesized using the same condition without TiO_2 and CNT sources, respectively.

Material Characterization: The crystal structure of the sample was investigated by XRD (Bruker with a Cu Kα X-ray source (λ =1.5418 Å). The morphology of the sample was collected by SEM and TEM. The surface chemical state of the sample was determined by XPS and Raman spectra. *The operando* synchrotron x-ray diffraction signals were collected at sector 17-BM of the

APS at Argonne National Laboratory. The beam size was 0.3 mm x 0.3 mm. The wavelength was 0.45165 Å. The obtained two-dimensional diffraction patterns were calibrated by a standard LaB6 sample and converted using the GSAS II package. A freestanding laminate was prepared from a mixture of CNT-MoS_2/TiO_2-C active materials, C45 and polytetrafluoroethylene with 8:1:1 wt% ratios. A modified 2032 cell with a 1.0 mm hole was used for in situ XRD tests. The cell was discharged and charged between 0.01 and 3 V at 0.3 C. Bruker Avance III spectrometer was used to examine 23Na magic-angle-spinning (MAS) NMR in a 14.1 T magnetic field with a 23Na Larmor frequency of 132.34 MHz. Electrodes were packed into 2.5 mm rotors and spun at a MAS rate of 25 kHz. The one-pulse sequence was employed, the recycle delay was 100 s, and the 90° pulse length was 2 μs. One M $NaCl_{(l)}$ with a ^{23}Na chemical shift at 0 ppm was used as the shift reference.

Electrochemical Measurements: The 2032-type coin cells were assembled using Na metal as a reference electrode and electrolyte of 1M $NaClO_4$ in the mixture of EC/PC (1:1). The working electrode was prepared by mixed as-prepared sampled, 10% super P and 10% CMC. The mass loading of active materials is 1.1-1.2 mg cm^{-2}. Galvanostatic charge-discharge measurements were conducted by a Land battery cycler. CV tests were performed at a scan rate from 0.1 to 2 mV s^{-1} using a CHI605D electrochemical workstation. EIS was recorded on AUTOLAB.

DFT Calculation: Density Functional Theory (DFT) calculations were carried

out with the gradient corrected exchange–correlation functional of Perdew, Burke, and Ernzerhof (PBE) under the projector augmented wave (PAW) method as implemented in the VASP code. The energy cut-off was set at 450 eV. As for periodic interface model construction, to minimize the lattice mismatch between

MoS_2 and TiO_2, a rather large slab containing p(10×10) MoS_2 and p(7×7) TiO_2 was adopted. All atoms were fully relaxed until the force on them was less than 0.01 eV $Å^{-1}$. Both spin-polarized and van der Waals (vdW) calculated by the DFT-D method of Grimme were taken into consideration. The band offset of the junction was obtained by alignment of the Fermi level, which was calibrated by the core level of sulfur.

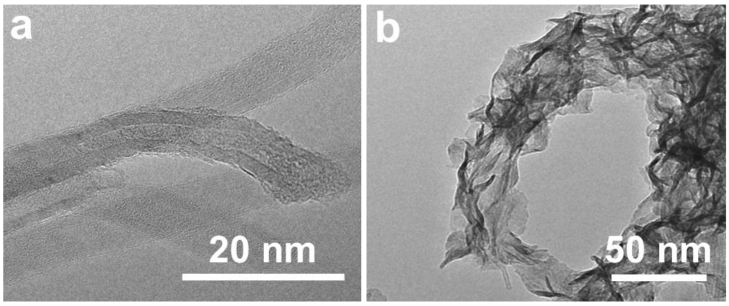

Figure S1. The morphology of a) pure CNT and CNT-MoS_2.

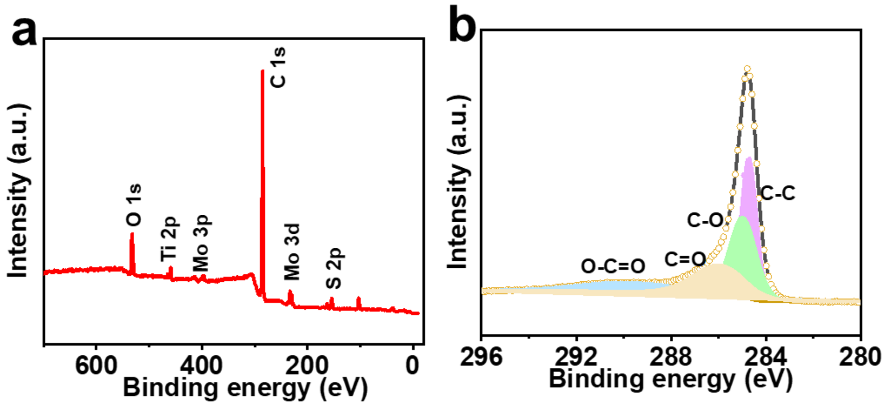

Figure S2. a) XPS spectrum and b) High-resolution XPS spectra of C 1s in the CNT-MoS_2/TiO_2-C.

The C 1s spectrum (Figure S2b, Supplementary Information) is resolved into four peaks at 284.3, 285.1, 285.2, and 290.2 eV, respectively. The strong peak at 284.3 eV can be assigned to the C-C, the latter three peaks are corresponding to the C-O, C=O, and O-C=O, respectively.

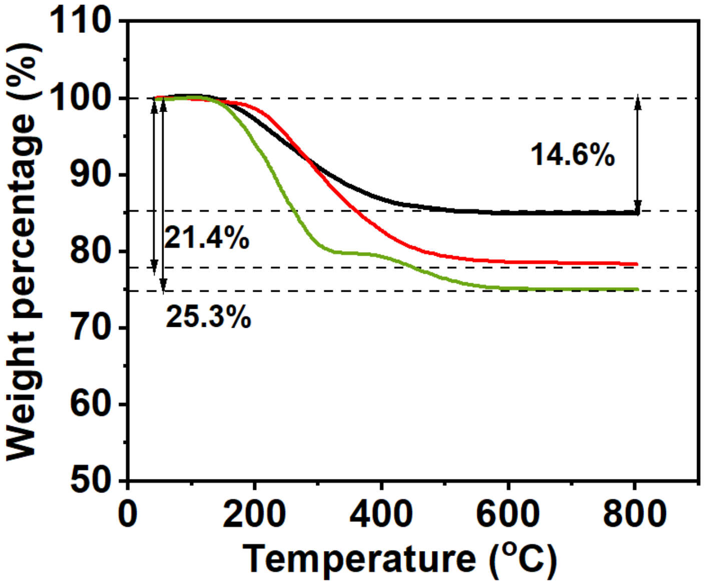

Figure S3. TGA profiles of MoS_2 (black line), CNT-MoS_2 (red line), and CNT-MoS_2/TiO_2-C (green line).

To further evaluate the carbon content in CNT-MoS_2/TiO_2-C composite, the thermogravimetric analysis (TGA) was conducted. As shown in Figure S3, the MoS_2 and CNT-MoS_2 are also tested to improve the accuracy of analysis. During the oxidation process, the MoS_2, CNT-MoS_2, and CNT-MoS_2/TiO_2-C samples exhibit 14.6, 21.4, and 25.3 % weight loss, respectively. The calculated content of the MoS_2/TiO_2 in CNT-MoS_2/TiO_2-C is 89.3%. And the amorphous carbon content is 3.9%.

The calculations for the mass ratio are shown below:

m_{CNT}=21.4%-14.6%=6.8%

$m_{CNT+carbon}$=25.3%-14.6%=10.7%

$m_{MoS2/TiO2}$=100%-10.7%=89.3%

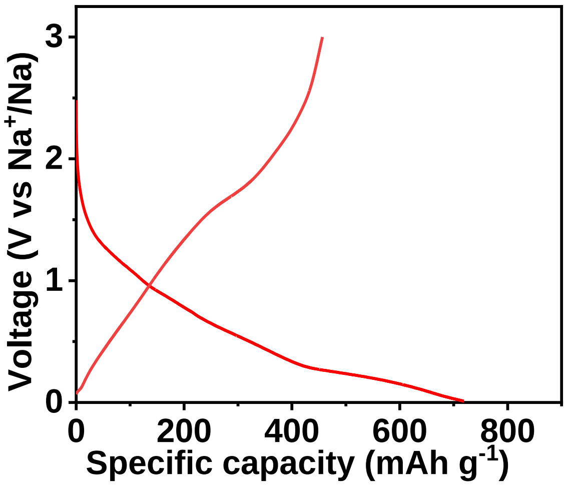

Figure S4. The charge-discharge profile of the CNT-MoS_2 electrode at a current of 0.2 A g^{-1}.

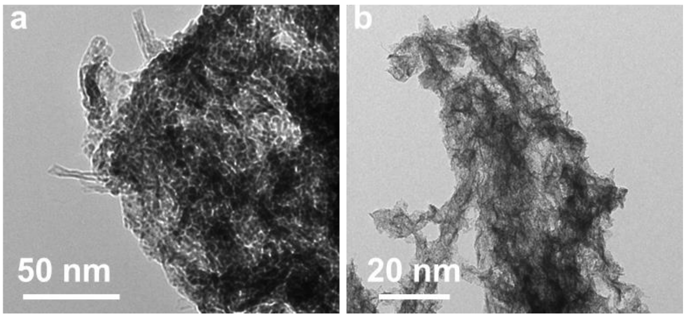

Figure S5. TEM images of CNT-MoS_2 electrode after 50 cycles at current of 1 A g^{-1}.

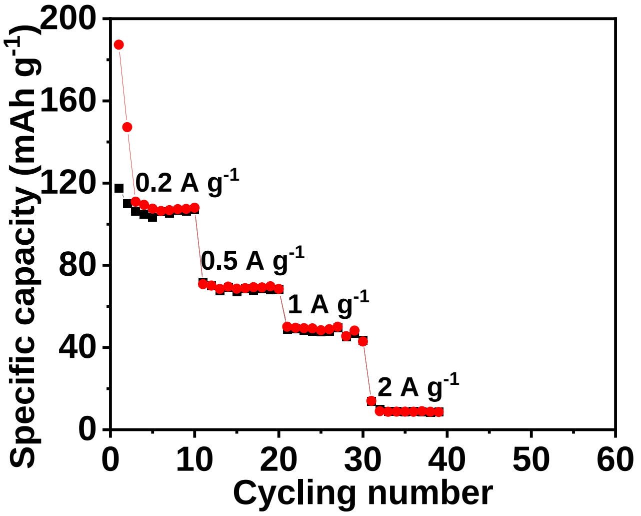

Figure S6. The rate capability of TiO_2-C electrode.

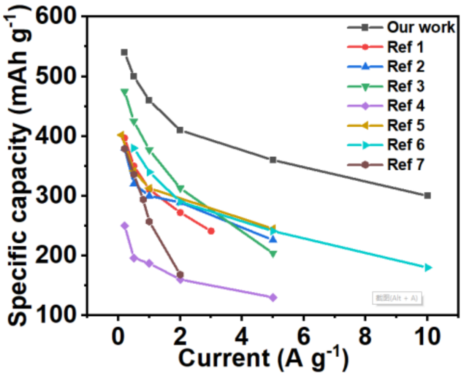

Figure S7. The comparison of the electrochemical performance of CNT-MoS_2/TiO_2-C electrode with electrode materials reported in literature.[1-7]

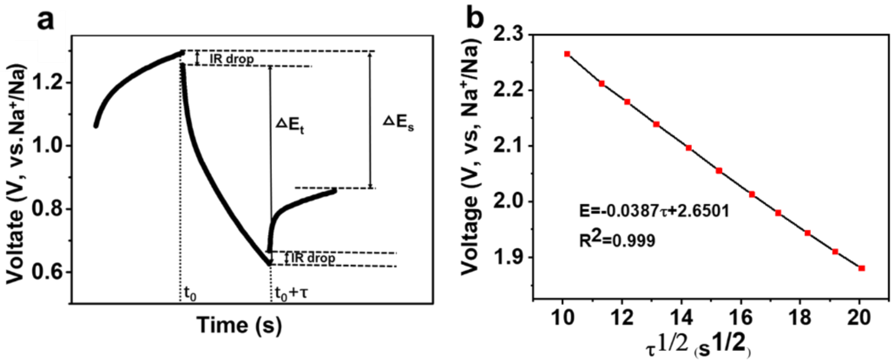

Figure S8. a) Schematic of GITT technique and b) Linear behavior of the E vs $\tau^{1/2}$ relationship.

The diffusion coefficient can be expressed as the following equation:

$$D_{Na+} = \frac{4}{\pi}\left(\frac{m_B V_M}{M_B A}\right)^2 \left(\frac{\Delta E_S}{\tau(dE_\tau)/d(\sqrt{\tau})}\right)^2 \quad (\tau \ll L^2/D_{Na+}) \qquad (1)$$

In this equation, τ(s) is the constant current flux time, m_B (g) is the active mass of the electrode, V_M (cm^3 mol^{-1}) is the molar volume of the electrode, M_B (g mol^{-1}) is the molecular weight, A (cm^2) is the surface area of the electrode, L (cm) is the thickness of the electrode, E_S (V) is the total change in cell voltage during a single step and $E\tau$ (V) is the voltage change in the steady state during a single step.

If E versus $\sqrt{\tau}$ shows a linear behavior during the current pulse, the equation can be transformed into:

$$D_{Na+} = \frac{4}{\pi}\left(\frac{m_B V_M}{M_B A}\right)^2 \left(\frac{\Delta E_S}{\Delta E_t}\right)^2 \quad (\tau \ll L^2/D_{Na+}) \qquad (2)$$

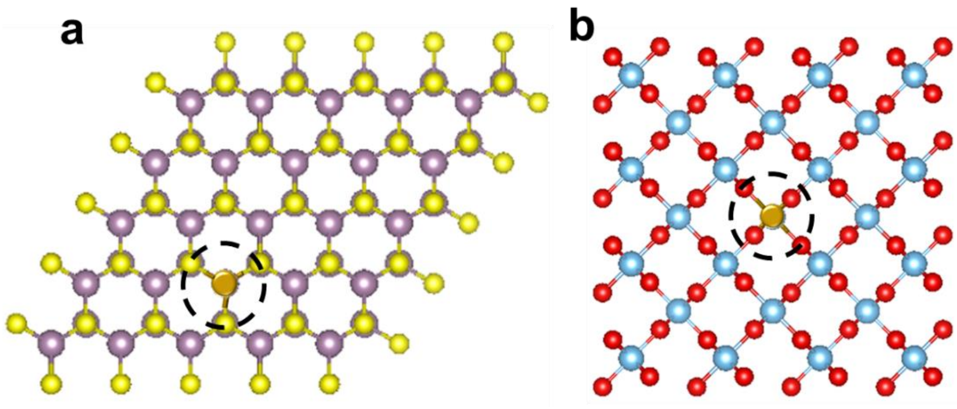

Figure S9. Na atom adsorption at a) MoS_2 and b) TiO_2 surface.

References:

1. K. Ma, H. Jiang, Y. Hu and C. Li, *Advanced Functional Materials*, 2018, 201804306.
2. Y. Li, R. Zhang, W. Zhou, X. Wu, H. Zhang and J. Zhang, *ACS Nano*, 2019, 9b00383.
3. M. Hou, Y. Qiu, G. Yan, J. Wang, D. Zhan, X. Liu, J. Gao and L. Lai, *Nano Energy*, 2019, 048.
4. G. Jia, D. Chao, N. H. Tiep, Z. Zhang and H. J. Fan, *Energy Storage Materials*, 2018, 019.
5. L. Jing, G. Lian, F. Niu, J. Yang, Q. Wang, D. Cui, C.-P. Wong and X. Liu, *Nano Energy*, 2018, 084.
6. J. Duan, G. Qin, L. Min, Y. Yang and C. Wang, *ACS Applied Materials & Interfaces*, 2018, 13570.
7. Z. Chen, D. Yin and M. Zhang, *Small*, 2018, 201703818.
